# Visual Verticality Perception in Spinal Diseases: A Systematic Review and Meta-Analysis

**DOI:** 10.3390/jcm9061725

**Published:** 2020-06-03

**Authors:** Esteban Obrero-Gaitán, Francisco Molina, Rafael Del-Pino-Casado, Alfonso Javier Ibáñez-Vera, Daniel Rodríguez-Almagro, Rafael Lomas-Vega

**Affiliations:** 1 Department of Health Sciences, University of Jaén, Paraje Las Lagunillas s/n, 23071 Jaén, Spain; eobrero@ujaen.es (E.O.-G.); ajibanez@ujaen.es (A.J.I.-V.); dralmagro4@gmail.com (D.R.-A.); rlomas@ujaen.es (R.L.-V.); 2 Department of Nursing, University of Jaén, Paraje Las Lagunillas s/n, 23071 Jaén, Spain; rdelpino@ujaen.es

**Keywords:** spinal diseases, idiopathic scoliosis, sensory integration, sense of verticality, subjective visual vertical, rod and frame test

## Abstract

Patients diagnosed with traumatic or non-traumatic spinal pain and idiopathic scoliosis frequently suffer from imbalance. The evaluation of the perception of verticality by means of visual tests emerges as a quick and easy tool for clinical management of the balance disorders. Several studies have assessed the visual perception of verticality in spinal diseases obtaining controversial results. The aim of our study is to analyze the perception of visual verticality in subjects with several spinal diseases in comparison with healthy subjects. A meta-analysis was carried out. PubMed MEDLINE, Scopus, WoS, CINAHL, and SciELO databases were searched until January 2020. The standardized mean difference (SMD) was calculated to analyze differences between patients and healthy controls. Fifteen studies with a total of 2052 patients were included. In comparison with healthy subjects, a misperception of verticality was found in patients with spinal pain when the perception of the verticality was assessed with the rod and frame test (SMD = 0.339; 95% confidence interval (CI) = 0.181, 0.497; *p* < 0.001). It seems that the perception of visual verticality is not altered in patients with idiopathic scoliosis (*p* = 0.294). The present meta-analysis shows a misperception of visual verticality only in patients with spinal pain.

## 1. Introduction

Spinal diseases include pathologies of the neck, thorax, and the lumbosacral area of the spine [1,2]. Spine-related disorders are among the most common problems in clinical medicine [3,4,5]. The last Global Burden of Disease Study reported that pain related to spinal diseases is the main cause of disability [6,7], work absenteeism [8], and socioeconomic burden worldwide [9]. In the United States, neck and back pain are the second cause for visits to physicians and cause high levels of disability and financial burden [10]. At least 80% of adults have experienced neck or low back pain at some point in their life [11]. In the United States, the economic burden of spinal diseases was 87.6 billion dollars in 2013, one of the highest after diabetes, stroke, and ischemic heart disease [12]. For these reasons, it is of paramount importance to establish effective and accessible treatments to promote health and reduce the negative consequences of spinal diseases [13,14]. 

In addition to pain, ocular and head motion impairments, dizziness, and unsteadiness are frequent complaints in patients with spinal diseases such as neck pain [15]. Balance impairment has been found to be common among patients with spinal diseases [16,17]. For example, a distortion of body balance and body postural control has been described in patients with a variety of neck injuries [18] probably due to a mismatch of the proprioceptive, visual, and vestibular afferent information needed to maintain proper balance [19]. The literature suggests that such postural alterations mainly appear in the acute phase of the condition, during the early stages of spinal diseases, and that they are valid predictors of a poor prognosis [20]. Furthermore, previous studies have established a possible relationship between idiopathic scoliosis and poor postural control [21,22], due to a hypothetical vestibular dysfunction or to alterations in the integration of sensorimotor information [23]. 

The perception of verticality involves the estimation of own-body verticality (subjective postural vertical, or SPV) and the verticality of objects appearing in the vision field (subjective visual vertical test, SVV; rod and frame test, RFT) or perceived by touch (haptic vertical) [24]. The SVV is the main test proposed to assess the perception of verticality in neurotological research and clinical practice [25]. SVV is a test that depicts one’s own body position in space with respect to gravity, and it is related with the subject’s ability to maintain balance and posture [26]. In this test, the subject adjusts a visible luminous line, in darkness, with the perceived direction of the earth vertical (the gravity line) without other visual references [27,28,29]. Another test designed to determine the perception of visual verticality is the RFT. In it, a rod is displayed, in darkness, inside a tilted or untilted frame with respect to the earth vertical [30]. Although both tests evaluate the visual perception of verticality, the SVV mainly assesses the vestibular contribution to the creation of the sense of verticality [31]. However, when the visual background is altered in the RFT by a tilted or untilted frame, proprioceptive and visual inputs are required to estimate visual verticality [32]. 

Finding out which potential alterations of visual verticality may occur in different spinal diseases would certainly shed light on the physiopathological mechanisms of these substantial and widespread health problems and open new therapeutic pathways for their treatment. For this reason, in recent years, interest has grown concerning the measurement of the perception of visual verticality in spinal diseases. However, studies looking into the matter have yielded contradictory results, probably due to methodological differences in the assessment and interpretation of the perception of visual verticality through RFT or SVV [33,34]. Therefore, the aim of our review and meta-analysis was to analyze the misperception of visual verticality in patients with several spinal pain and deformity diseases in comparison with healthy subjects.

## 2. Methods

### 2.1. Protocol

This meta-analysis was conducted according to the Preferred Reporting Items for Systematic Reviews and Meta-Analyses statement (PRISMA) [35] and the recommendations of the Meta-Analysis of Observational Studies in Epidemiology (MOOSE) group [36].

### 2.2. Data Sources and Search Strategy

Two authors (E.O.-G. and R.L.-V.) independently carried out the bibliographic search between 26 December 2019 and 30 January 2020. A bibliographic search was performed for relevant literature in PubMed, MEDLINE, Scopus, Web of Science, CINAHL, and SciELO as well as in the reference lists of the full-text articles retrieved and other reviews. The keywords used were “verticality sense”, “spinal diseases”, “subjective visual vertical”, and “rod and frame test”. Medical subject headings (MeSH) and keyword bases in main studies were searched using the combination proposed in each database with the appropriate tags. No language and publication date restrictions were used. Table 1 shows the search strategies for the different databases. 

### 2.3. Study Selection and Inclusion Criteria

Two blinded reviewers (E.O.-G. and R.L.-V.) independently screened the titles and abstracts. Each article was examined in detail if at least one of the researchers selected it during the inclusion phase based on the title or abstract. Discrepancies that arose during full-text screening were resolved by a third researcher (F.M.).

The inclusion criteria used were as follows: (1) observational studies, including cross-sectional, case–control, and cohort studies; (2) studies that measure the ability of patients with spinal diseases to estimate static SVV in the sitting or upright position; (3) studies that use the SVV test and/or the RFT with tilted and untilted frame with respect to earth vertical; and (4) studies that included a comparison groups of healthy subjects. The exclusion criteria proposed were as follows: (1) studies that assess the perception of visual verticality after any therapeutic intervention; (2) observational studies with only one group and (3) studies not susceptible to obtain the absolute error mean and its standard deviation with validated procedures according to the *“Cochrane Handbook for Systematic Reviews of Interventions”* [37] and the recommendations of Hozo et al. (2005) [38].

### 2.4. Data Extraction

Two independent reviewers (E.O.-G. and D.R.-A.) collected the data of the exposed and non-exposed groups of each study using a standardized data-collection form. Disagreements were solved by consensus with a third reviewer (R.L.-V.). The characteristics recorded of each study selected for the review were the research design; authorship and publication date; total sample size; the number of participants in the exposed (patients diagnoses of a spinal disease) and non-exposed group (healthy individuals); age; sex (male or female); type of spinal disease such as spinal pain (neck or low back pain) or musculoskeletal spinal deformity (idiopathic scoliosis); and the phase of progression since the onset (acute or chronic). The primary outcome was the perception of visual verticality using the absolute error mean and its standard deviation (SD) measured with SVV test or RFT in the exposed and healthy subjects. Other error calculation measurements of the RFT, as the variability of the error (VE), were not included in our meta-analysis due to their limited use in the scientific literature [39]. When a study met the inclusion criteria and did not provide the SD necessary for the statistical analysis, we collected the standard error mean, ranges (minimum and maximum), interquartile ranges, and the sample size to be transformed into in SD, according to the “*Cochrane Handbook for Systematic Reviews of Intervention*” [37]. Only values obtained from the SVV test and RFT were analyzed in the quantitative synthesis. Data from the selected studies were extracted at baseline without any therapeutic intervention on the control or exposed groups.

### 2.5. Quality Assessment

The Newcastle–Ottawa Scale (NOS) [40] was used to assess the methodological quality of the studies included in our meta-analysis. Three criteria are included in the NOS checklist: study selection (maximum, 4 stars), comparability (maximum, 2 stars) and assessment of outcome/exposure (maximum, 3 stars) [41]. Quality scores ranged from 0 (lowest) to 9 stars (highest) [42]. The NOS score classifies the studies as low quality (score 1–3), moderate quality (score 4–6), or high quality (score 7–9) [40,43]. Following the recommendations of Meader (2014) [44] and the Grading of Recommendations Assessment, Development, and Evaluation (GRADE) system [44], inconsistency, imprecision, and risk of publication bias were also evaluated. Inconsistency was evaluated through heterogeneity of findings in individual studies [45], and imprecision was assessed through the number of included studies (large: >10 studies; moderate: 5–10 studies; and small: <5 studies) and the median sample size (high: >300 participants; intermediate: 100–300 participants; and low: <100 participants) [37,44].

Two independent reviewers (A.J.I.-V. and F.M.) assessed the quality of included studies, and a third author (R.L.-V.) resolved the possible disagreements.

### 2.6. Statistical Analysis

The statistical software Comprehensive Meta-Analysis (Version 3.3.070 Biostat, Englewood, NJ, USA) [46] was used to perform the meta-analysis. Two authors carried out the quantitative synthesis (R.D.-P.-C and E.O.-G.). According to the recommendations of Cooper (2009) [47], the random-effects model of DerSimonian and Laird [48] was used to estimate the pooled effect and its 95% confidence interval (CI) with the aim to improve the generalization of the findings. The pooled effect was calculated as a standardized mean difference (SMD) [37,49]. The magnitude of the SMD can be interpreted in three effect strength levels: small (SMD = 0.2); medium (SMD = 0.5); and large (SMD = 0.8) [49,50]. Forest plots were used to display our findings [51]. The potential asymmetry and publication bias were visually evaluated by funnel plot [52,53], and the adjusted pooled effect was estimated (taking into account any possible publication bias) by the trim-and-fill method [54,55]. Heterogeneity was analyzed using Cochran’s Q test [56] and *I*^2^ statistic of Higgins [45] (where <25% indicates slight heterogeneity; 25%–50%, moderate heterogeneity; and ≥50%, high heterogeneity). Spinal pain diseases and idiopathic scoliosis were analyzed independently in three meta-analyses according to the test employed to measure the subjects’ perception of verticality in comparison to healthy controls, given the specific characteristics of each.

## 3. Results

### 3.1. Study Selection

**Figure 1:**
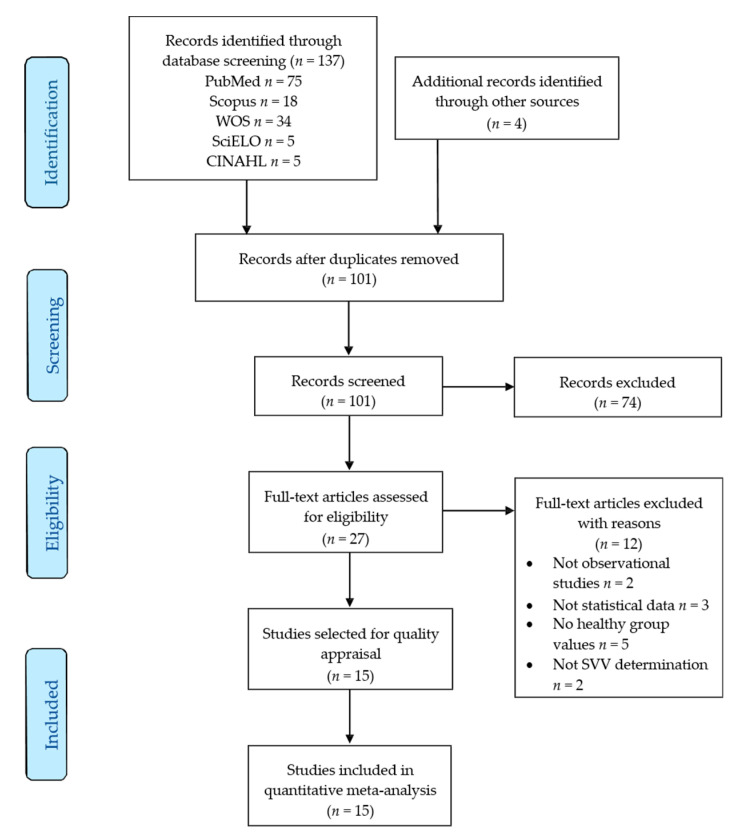
Preferred Reporting Items for Systematic Reviews and Meta-Analyses (PRISMA) flow chart.

Information concerning the bibliographic search and study selection process is displayed in the PRISMA flow chart (Figure 1). Based on the search criteria, 137 references were retrieved from the different databases and 4 additional records were identified in the lists of references of other articles. After removing duplicates, 101 records were reviewed. Of these, 74 studies were deleted for not being relevant by title and abstract and 12 records were rejected for not meeting the inclusion criteria. Figure 1 shows the number of records removed and the reasons for their exclusion. Finally, 15 studies [57,58,59,60,61,62,63,64,65,66,67,68,69,70,71] were included in this review.

### 3.2. Characteristics of Studies Included in the Meta-Analysis

The main characteristics of the studies analyzed are shown in Table 2. The selected studies including 11 studies with 14 samples of patients with spinal pain [57,58,59,61,62,63,64,68,69,70,71] and 4 studies with 4 samples of idiopathic scoliosis patients [60,65,66,67]. A total of 31 independent comparisons were identified. The total number of participants included in the selected studies was 2052 (mean age: 41.73 ± 16.11 years old). The mean number of participants per study was 67.48. At first, results were grouped in three overall meta-analyses. Two meta-analyses were performed for subjects with spinal pain examined using SVV or RFT. For idiopathic scoliosis, only SVV data were available.

### 3.3. Quality Assessment of Studies Included in the Meta-Analysis

The methodological quality of the selected studies, assessed by the Newcastle–Ottawa Scale (NOS), was low-to-moderate (mean quality: 4). Six studies [58,62,63,66,68,71] (40% of the selected references) were deemed low quality and the remaining 9 studies [57,59,60,61,64,65,67,69,70] (60% of included studies) were considered of moderate quality. Table 3 shows the NOS rating for selection, comparability, and exposure/outcome of the studies included in this review.

### 3.4. Results of the Overall Meta-Analysis about Perception of Visual Verticality in Spinal Pain Patients Measured with the SVV Test

**Figure 2:**
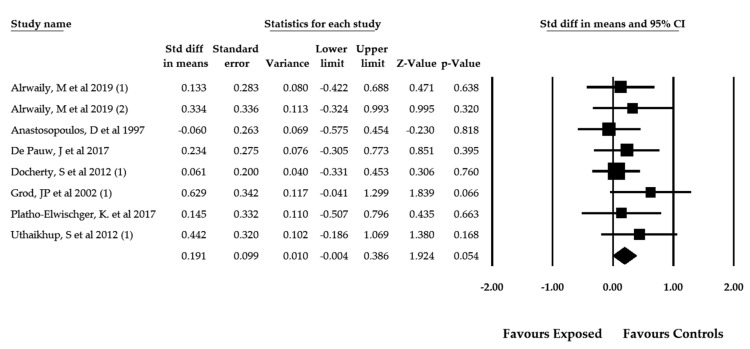
Forest plot for the perception of the visual verticality assessed with the SVV test in patients with spinal pain (effect sizes are expressed as standardized mean difference).

In 7 studies [57,58,61,63,68,70,71] with 8 independent comparisons, the misperception of visual verticality was measured using the SVV test. These studies included 459 patients (74% female and 26% male with a mean age of 49.75 ± 11.80 years old) with spinal pain, whether neck pain [58,61,63,68,70,71] or low back pain [57] (Table 4). The pooled effect (SMD = 0.199; 95% CI = −0.04, 0.386; *p* = 0.056) revealed that patients with spinal pain, as measured by the SVV test and regardless of pain locus, did not exhibit signs of misperceiving visual verticality with respect to the earth vertical (Figure 2). The funnel plot was asymmetric (Figure S1), and the adjusted pooled effect calculated with the trim-and-fill method (adjusted SMD = 0.150) suggested a risk of publication bias. There was no heterogeneity among studies (*I*^2^ = 0%), and the number of participants per study (57.37) suggests that results have a low precision level.

### 3.5. Results of the Overall Meta-Analysis about Perception of Visual Verticality Measured with RFT in Patients with Different Spinal Pain

**Figure 3:**
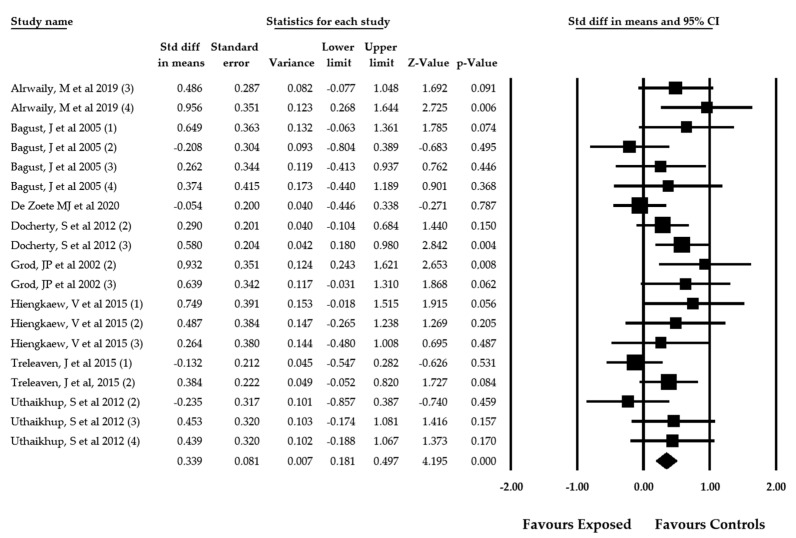
Forest plot for the perception of the visual verticality assessed with the RFT in patients with spinal pain (effect sizes are expressed as standardized mean difference).

When RFT was used to assess the perception of visual verticality in patients with spinal pain, 8 studies [57,59,62,63,64,69,70,71] with 19 independent comparisons were selected to perform the meta-analysis. A total of 1308 individuals (69% female and 31% male with a mean age of 44.17 ± 13.12 years old) with spinal pain were reported in these articles. The participants of 7 studies [59,62,63,64,69,70,71] had been diagnosed with neck pain, and only one study concerned subjects with low back pain [57] (Table 4). The pooled effect size (SMD = 0.339; 95% CI = 0.181, 0.497; *p* < 0.001) signaled a moderate misperception of visual verticality in patients with spinal pain assessed with RFT (Figure 3). The funnel plot was clearly asymmetric and a certain risk of publication bias was detected (Figure S2). This was later confirmed by the calculation of the adjusted pooled effect through the trim-and-fill method. The adjusted SMD was 0.230, which suggests that the original pooled effect could be overestimated by 33%. Heterogeneity was not present in this meta-analysis (*I*^2^ = 0%), and the number of participants per study (68.84) suggested a low precision level.

### 3.6. Results of the Overall Meta-Analysis about Perception of Visual Verticality in Patients with Idiopathic Scoliosis

**Figure 4:**
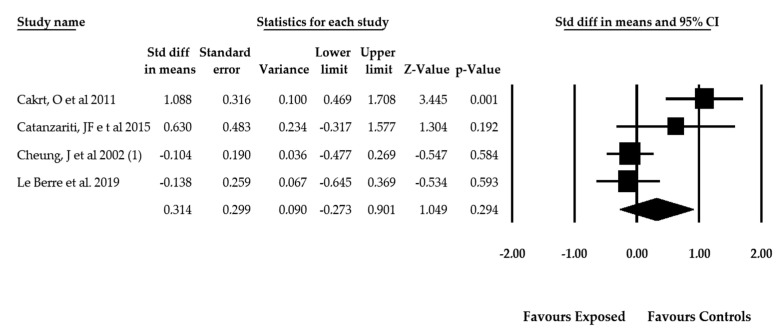
Forest plot for the perception of the visual verticality assessed with the SVV in patients with idiopathic scoliosis (effect sizes are expressed as standardized mean difference).

A total of 4 studies [60,65,66,67] with 4 independent comparisons reported SVV test data from 285 participants (238 females and 37 males with a mean age of 14.48 ± 0.37 years old). Their pooled effect (SMD = 0.314; 95% CI = −0.273, 0.901; *p* = 0.294) was not statistically significant (Table 4). It may therefore be concluded that patients with idiopathic scoliosis subject to the SVV test do not exhibit signs of misperception of the visual verticality in comparison with healthy subjects (Figure 4). No studies employed RFT to determine perception of visual verticality. Publication bias was not considered given that the funnel plot was symmetric (Figure S3) and the adjusted pooled effect, calculated with the trim-and-fill method, did not differ from the original. Heterogeneity was very slight (*I*^2^ = 2%), and the number of participants per study was 71.25, revealing a low precision level.

## 4. Discussion

This is the first systematic review with meta-analysis to look into the alteration of the visual perception of verticality in patients with spinal diseases. The assessment of the perception of visual verticality in research and clinical practice is frequently carried out by practitioners [72]. There are two tools that are commonly used to measure the visual perception of verticality: The subjective visual vertical test (SVV) and the rod and frame test (RFT). Different studies looking into perception of visual verticality have revealed that it is impaired in neurological disorders such as stroke [73,74,75], Parkinson’s disease [76,77], multiple sclerosis [78], vestibular dysfunctions [79,80,81], chronic dizziness [82], and type II diabetes mellitus [83], among others.

The sense of verticality is a complex construct of the central nervous system (CNS) created from the visual, vestibular, and proprioceptive inputs of the neck and eye muscles [84,85]. A failure in one or more of these information systems could produce a misperception of verticality and result in related balance disorders [86,87]. Several studies have detected balance impairment in patients with spinal pain, especially those experiencing neck pain derived from traumatic and non-traumatic neck lesions [18,19,88]. This distorted balance has been related to alterations in somatosensory cervical inputs in the absence of other vestibular pathologies [89].

Our results confirm an impairment of the visual verticality perception in spinal pain patients when they were assessed with RFT. It is important to highlight that the RFT evaluates the contribution of visual and proprioceptive afferent information to the development of the sense of visual verticality [57], while SVV mainly analyzes the weight of vestibular cues in the perception of verticality [31,90]. For this reason, our findings could be interpreted as a specific failure in integrating visual and proprioceptive inputs in order to generate a perception of visual verticality in patients with spinal pain.

Previous neurophysiological studies have explained the relationship between visual inputs and spinal proprioceptive information for the creation of a perception of verticality [75]. Mechanoreceptors from the neck establish central and reflex connections with the vestibular, visual, and sympathetic systems [91]. Some reflexes, such as the cervico-collic reflex (CCR), the cervico-ocular reflex (COR), and the tonic neck reflex (TNR) are required to exercise precise control of head and eye movements and to maintain postural stability [92]. Along these lines, studies performed in animals or humans without vestibular dysfunctions have detected an increase in the COR after damage was inflicted in experimental conditions to the neck proprioceptive inputs [93,94]. Moreover, observational studies have described distorted oculo-motor control, disturbances in gaze stability, and alterations in head eye coordination, vergence, saccades, and ocular reflex activity in patients with traumatic neck pain or myofascial neck pain [95]. Taking this together, we suggest that the misperception of visual verticality and related balance disorders observed in patients with neck pain mainly derived from the cervico-ocular pathways alteration, but not from cervico-vestibular impairments. Following the recommendations of Grod and Diakow [71], Bagust [64], and Docherty [70], we believe that RFT should be used to assess the perception of visual verticality in patients with spinal pain in order to explore the relationship between neck pain and vertical discernment.

The present meta-analysis also analyzed the perception of visual verticality in idiopathic scoliosis. The hypothetic relationship between the function of the vestibular pathways and scoliosis development comes from animal and human studies. In animal experimental studies, the researchers induced spinal deformities after surgical damage on the vestibular apparatus [96,97]. These studies performed in *Xenopus laevis* larvae have described both a decrease in the number of fibers on the ipsilesional VIIIth nerve and a loss in the number of neurons in the ipsilesional tangential vestibular nucleus (TAN) after the experimental unilateral labyrinthectomy (UL). This cell death mainly affects the vestibulospinal neurons in the TAN. It is worth mentioning that TAN is the main graviceptive relay vestibular nucleus, and it is closely related to the vestibulo-ocular reflex (VOR) [98]. The loss of neurons in this nucleus after UL could provoke a bilateral imbalance of descending activity and spinal motoneuronal drive and may cause an irreversible asymmetric tone of axial and limb muscles. In consequence, the scoliotic deformity observed in this amphibian model may be the result of the permanent muscle tone imbalance. Since the TAN has a similar function to that of the mammals Deiter’s nucleus [99], the authors of these studies suggest that a previous alteration in the vestibular apparatus could provoke a spinal deformation in scoliosis patients. Moreover, some genome-wide studies [100,101] have detected in patients with scoliosis a single nucleotide polymorphism in genome regions that code for proteins implicated in vestibular development (CHL 1) [100] and vestibular function (Cntnap2) [102].

Misperception of visual verticality in patients with idiopathic scoliosis has only been measured with the SVV test. Our results demonstrate that patients with idiopathic scoliosis do not show a misperception of visual verticality when the contribution of vestibular inputs was analyzed. We suggest that the spinal deformity observed in patients with idiopathic scoliosis could be an adaptive response to a vestibular dysfunction [97]. Our hypothesis is that a previous misperception of the visual verticality due to vestibular impairment during the infant stage would force the patients to reach an inclined-rotated spine and head position in which a correct estimation of the visual verticality may be performed.

Some limitations must be considered regarding this meta-analysis. Firstly, some studies found in our bibliographic search were not included in the quantitative synthesis for failing to report the SVV or RFT values of the control group. Secondly, the low number of published studies reporting valid data for SVV or RFT reduces the precision of our meta-analysis. Moreover, future studies should determine the error of the SVV or RFT using more sensitive calculations such as the variability of the error (VE) instead of the absolute mean error or the constant error. Yet another limitation was that, in the studies considered here, the misperception of visual verticality in idiopathic scoliosis was not estimated by means of RFT. Due to this, the visual contribution to the perception of verticality and its relationship with spinal proprioception has yet to be explored for patients with idiopathic scoliosis. Before any of our findings are generalized, we must also take into account the limitations related with publication bias, although heterogeneity was not present. Future research should use RFT to examine patients with idiopathic scoliosis in order to add to the understanding of how the visual and proprioceptive systems contribute to the development of idiopathic scoliosis.

## 5. Conclusions

The present meta-analysis evaluated the relationship between spinal diseases and the perception of visual verticality. Patients with spinal pain diseases showed a misperception of visual verticality measured when examined using RFT. When the contribution of the vestibular inputs to the sense of verticality was analyzed in spinal diseases using the SVV test, subjects with spinal pain and idiopathic scoliosis did not show a misperception of visual verticality. In patients with spinal diseases, misperception of visual verticality seems to be related to visual and proprioceptive disturbances rather than to vestibular impairments.

## Figures and Tables

**Table 1:** Bibliographic search strategy.

Databases	Bibliographic Search Strategy
**MEDLINE PubMed**	(sensory integration (tiab) OR vertical perception (tiab) OR vertical sense (tiab) OR subjective visual vertical (tiab) OR subjective visual vertical test (tiab) OR subjective visual vertical perception (tiab) OR “rod and frame” (tiab)) and (spine (mh) OR spine (tiab) OR musculoskeletal disease (mh) OR musculoskeletal diseases (tiab) OR spinal diseases (mh) OR spinal diseases (tiab) OR spinal curvatures (mh) OR spinal curvatures (tiab) OR neck pain (mh) OR neck pain (tiab) OR neck injuries (mh) OR neck injuries (tiab) OR scoliosis (mh) OR scoliosis (tiab) OR adolescent idiopathic scoliosis (tiab) OR low back pain (mh) OR low back pain (tiab)
**Scopus**	(TITLE-ABS-KEY ((“subjective visual vertical” OR “perception of verticality” OR “visual verticality” OR “verticality sense” OR “rod and frame”)) AND TITLE-ABS-KEY ((“spinal pain” OR “spinal diseases” OR “musculoskeletal disorders” OR “neck pain” OR “neck injuries” OR “idiopathic scoliosis” OR “low back pain”)))
**Web of Science**	TOPIC:((*subjective visual vertical* OR *perception of verticality* OR *visual verticality* OR *rod and frame*)) AND TOPIC: ((*spinal pain* OR *spinal diseases* OR *musculoskeletal disorders* OR *neck pain* OR *neck injuries* OR *idiopathic scoliosis* OR *low back pain*))
**SciELO**	(subjective visual vertical or verticality perception or verticality or verticality sense)
**CINAHL**	(AB perception of verticality OR AB verticality sense OR AB subjective visual vertical OR AB visual vertical OR AB “rod and frame”) AND (AB musculoskeletal diseases OR AB neck pain OR AB idiopathic scoliosis OR AB low back pain)

**Table 2:** Main characteristics of the studies included in the meta-analysis.

Study____________________				Exposed Group					Control Group		
Author and Year	Design	VV Measure	N	N_exp_	Age (mean)	Gender (M/F)	Pathology	Onset	N_c_	Age (mean)	Gender (M/F)
Alrwaily et al. 2020 [57]	CC	SVV/ RFT	64	39	42.4	14/27	LBP	C	25	34	8/17
Anastasopoulos et al. 1997 [58]	CC	SVV	58	29	50.2	NR	NP	C	29	45.5	NR
Bagust et al. 2005 [64]	CC	RFT	88	71	38.97	24/47	NP	C/A	17	33.6	7/10
Cakrt et al. 2011 [65]	CC	SVV	46	23	14.5	5/18	IS	C	23	14	5/18
Catanzariti et al. 2015 [66]	CC	SVV	40	35	14.2	4/31	NP	C	5	14.9	5/0
Cheung et al. 2002 [67]	CH	SVV	129	89	15	15/74	IS	C	40	12	8/32
De Pauw et al. 2017 [68]	CC	SVV	54	24	59.2	4/20	NP	C	30	59.4	12/18
De Zoete et al. 2020 [69]	CC	RFT	100	50	35.5	20/30	NP	C	50	NR	23/27
Docherty et al. 2012 [70]	CC	SVV/ RFT	100	50	48.1	10/40	NP	C	50	47.9	10/40
Grod et al. 2002 [71]	CH	SVV/ RFT	36	19	38.5	8/11	NP	A	17	38.6	10/7
Hiengkaew et al. 2014 [59]	CC	RFT	28	14	44,4	3/11	NP	C	14	43,6	7/7
Le Berre et al. 2019 [60]	CC	SVV	60	30	14.2	30/0	IS	C	30	13.9	30/0
Platho-Elwischger et al. 2017 [61]	CC	SVV	43	30	59	11/19	NP	C	13	52,8	4/9
Treleaven et al. 2015 [62]	CC	RFT	126	78	33,5	31/47	NP	C	48	29,4	13/35
Uthaikhup et al. 2012 [63]	CC	RFT	40	20	73,2	6/14	NP	C	20	69,5	8/12

VV measure: Visual Vertical measure; CC: case–control studies; CH: cohort studies; SVV: subjective visual vertical test; RFT: rod and frame test; N: sample size; N_exp_: cases sample size; M: male; F: female; LBP: low back pain; NP: neck pain; IS: idiopathic scoliosis; C: chronic; A: acute; N_c_: controls sample size; NR: not reported data.

**Table 3:** Newcastle–Ottawa Scale (NOS) score for methodological quality assessment of included studies.

Study	S1	S2	S3	S4	C1	E1	E2	E3	Total
Alrwaily et al. 2020 [57]	-	*	*	*	**	-	*	-	6
Anastasopoulos et al. 1997 [58]	-	-	-	-	*	-	-	-	1
Bagust et al. 2005 [64]	-	-	*	*	**	-	*	*	6
Cakrt et al. 2011 [65]	*	-	*	*	**	*	-	-	6
Catanzariti et al. 2015 [66]	-	-	-	-	-	-	-	*	1
Cheung et al. 2002 [67]	-	*	*	*	*	-	-	-	4
De Pauw et al. 2017 [68]	-	-	-	*	*	*	-	-	3
De Zoete et al. 2020 [69]	-	-	*	*	*	-	*	-	4
Docherty et al. 2012 [70]	-	-	*	*	**	-	-	*	5
Grod et al. 2002 [71]	-	-	-	-	*	-	-	-	1
Hiengkaew et al. 2014 [59]	-	-	*	*	**	-	-	*	5
Le Berre et al. 2019 [60]	-	*	-	*	*	-	*	*	5
Platho-Elwischger et al. 2017 [61]	-	*	*	*	**	-	-	*	6
Treleaven et al. 2015 [62]	-	-	-	*	*	-	-	*	3
Uthaikhup et al. 2012 [63]	-	-	*	*	-	-	*	-	3

Each study can be awarded a maximum of one star for each numbered item within the Selection (S) and Exposure (E) categories. A maximum of two stars can be given for Comparability (C). S1: adequate case definition; S2: representativeness of the cases; S3: selection of controls; S4: definition of controls; C1: comparability of cases and controls; E1: ascertainment of exposure; E2: same method of ascertainment for cases and controls; E3: non-response rate.

**Table 4:** Summary of findings in the meta-analyses.

				Effect Size			Publication Bias			Heterogeneity	
Group	K	N	N_s_	SMD	95% CI	*p-*Value	Funnel Plot	Trim and Fill		*q*-Value	*I* ^2^
								Adjusted SMD	% of Change		
**SVV in spinal pain patients**	8	459	57.37	0.191	−0.040; 0.386	0.054	Asym.	0.150	22%	3.857	0%
**RFT in spinal pain patients**	19	1308	68.84	0.339	0.181; 0.497	0.000	Asym.	0.248	28%	16.124	0%
**SVV in** **IS patients**	4	325	81.25	0.314	−0.273; 0.901	0.294	Sym.	0.314	0%	3.061	2%

SVV: subjective visual vertical test; RFT: rod and frame test; IS: idiopathic scoliosis; K: number of studies; N: number of participants in each meta-analysis; N_s_: number of participants per study; SMD: standardized mean difference; 95% CI: confidence interval 95%; Asym: asymmetric funnel plot; Sym: symmetric funnel plot; *I*^2^: degree of inconsistency of Higgins.

## Supplementary Materials

The following are available online at https://www.mdpi.com/2077-0383/9/6/1725/s1, Figure S1: Funnel plot of the meta-analysis of patients with spinal pain assessed with SVV test; Figure S2: Funnel plot of the meta-analysis of patients with spinal pain assessed with RFT; and Figure S3: Funnel plot of the meta-analysis of patients with idiopathic scoliosis assessed with SVV test.
