# Supplementary figures and images for: Visual Verticality Perception in Spinal Diseases: A Systematic Review and Meta-Analysis
Source: J Clin Med. 2020 Jun 3;9(6):1725. doi: 10.3390/jcm9061725 (PMC7356295; doi:10.3390/jcm9061725)

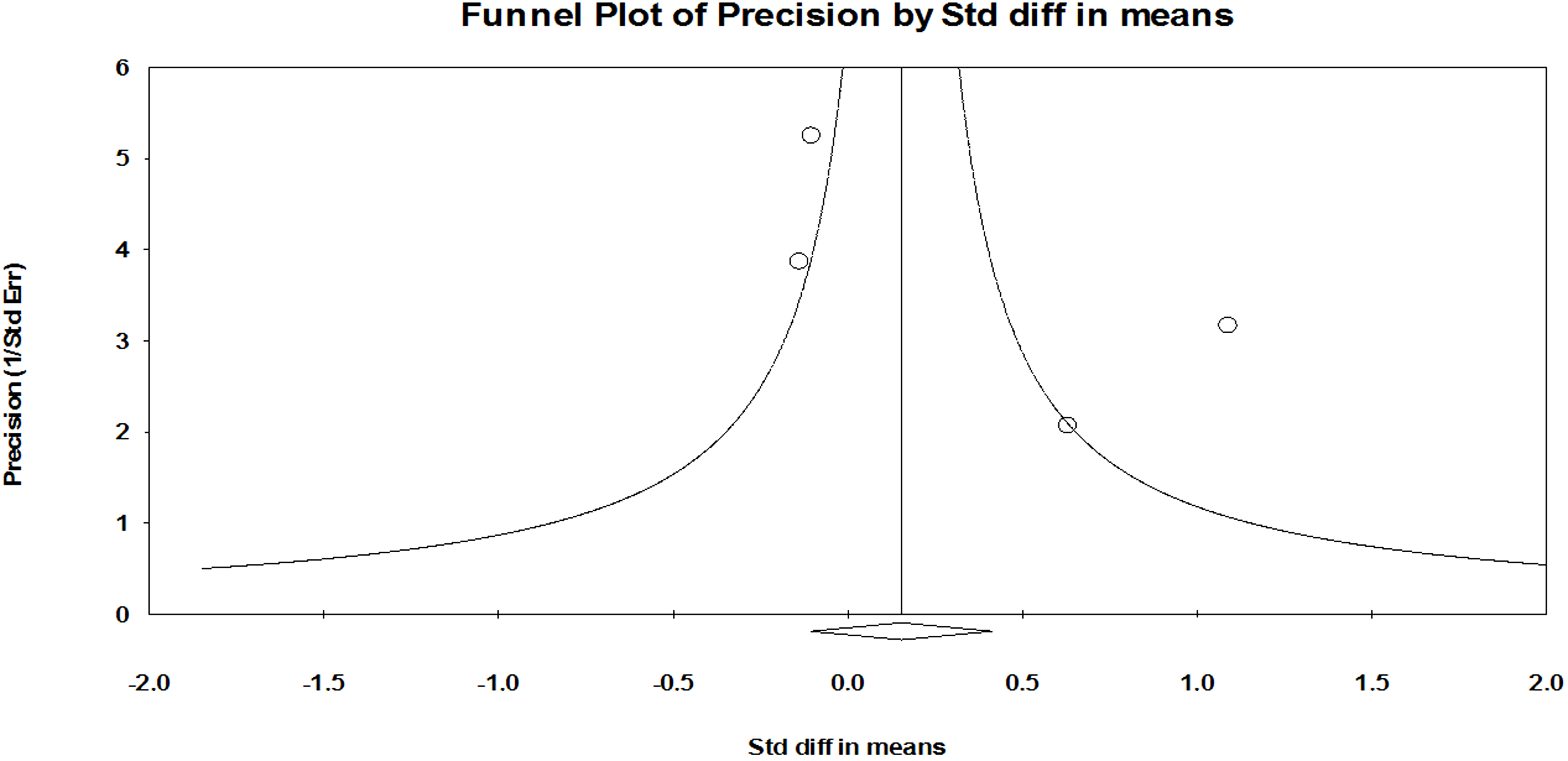

Supplement: Supplementary file 1 [file jcm-09-01725-s001.zip › Figure S3 Funnel plot scoliosis.tif]

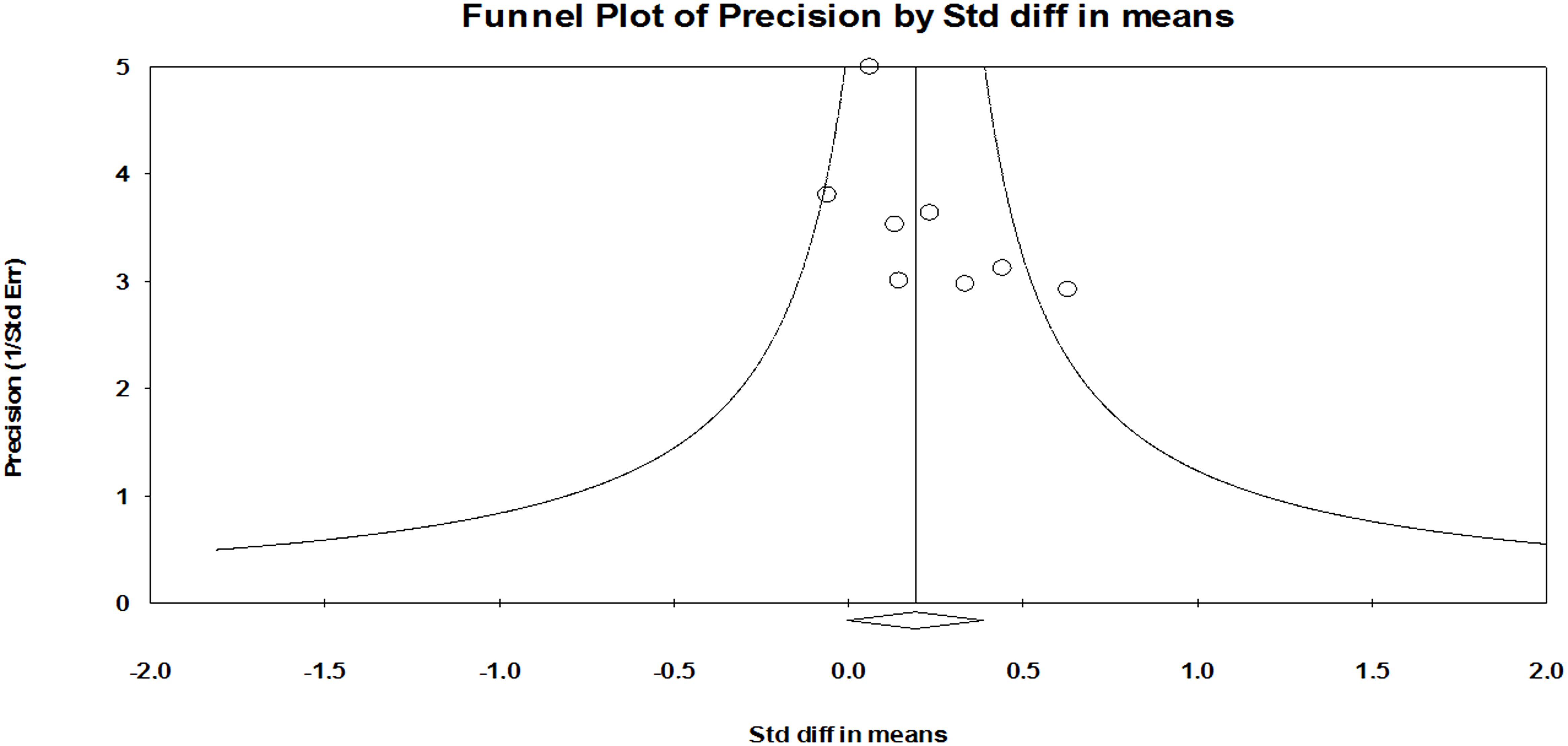

Supplement: Supplementary file 1 [file jcm-09-01725-s001.zip › Figure S1 Funnel plot SVV spinal pain.tif]

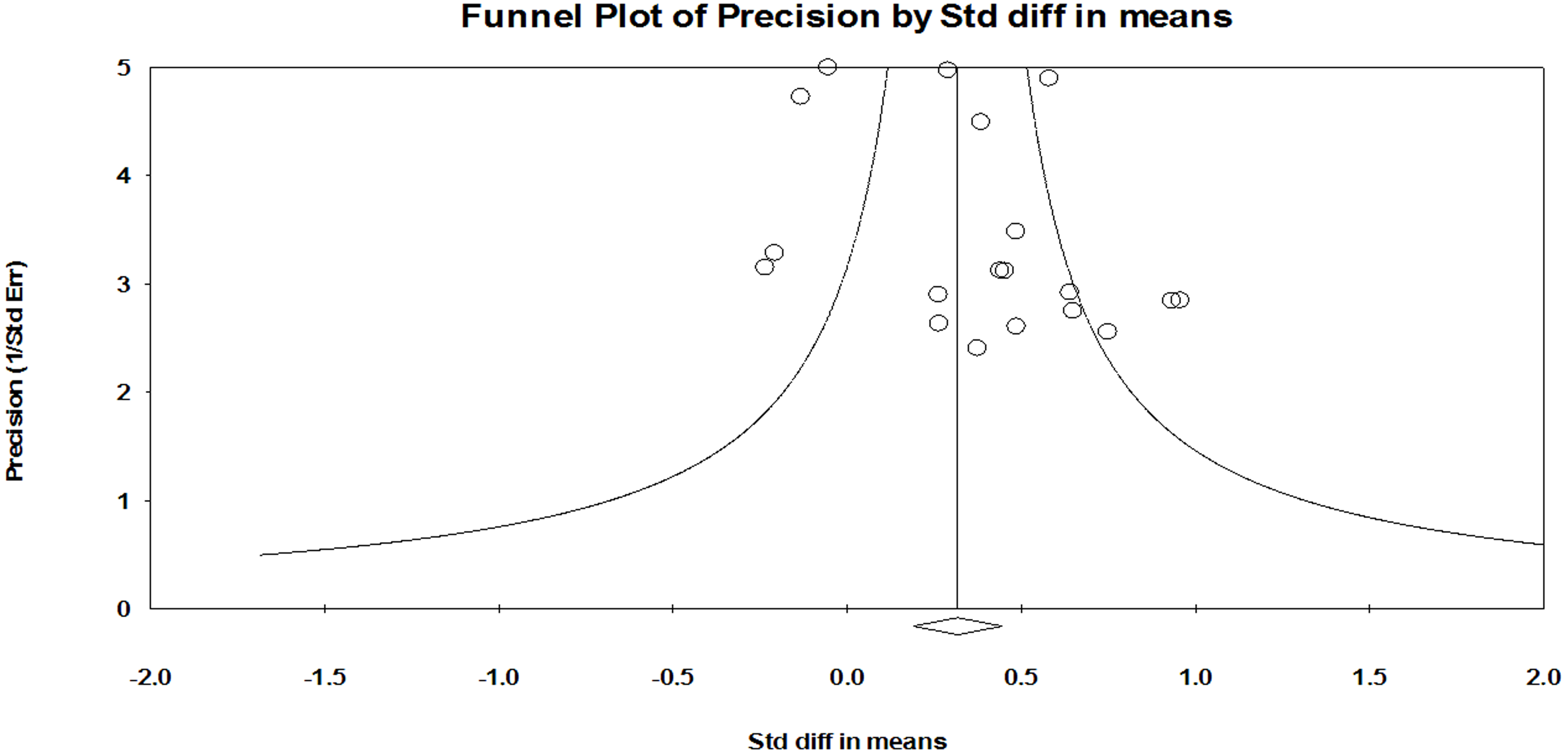

Supplement: Supplementary file 1 [file jcm-09-01725-s001.zip › Figure S2 Funnel plot RFT spinal pain.tif]
